# Directrices estructurales para la sostenibilidad del plan de control de infecciones de tuberculosis en instituciones prestadoras de servicios de salud, distrito de Cali, 2021

**DOI:** 10.7705/biomedica.8210

**Published:** 2026-08-20

**Authors:** Mónica Espinosa, Lucy del Carmen Luna, Claudia Rojas, Gloria Mercedes Puerto, Robinson Pacheco, Claudia Marcela Castro

**Affiliations:** 1 Grupo de Micobacterias, Dirección de Investigación en Salud Pública, Instituto Nacional de Salud, Bogotá, D. C., Colombia Instituto Nacional de Salud Bogotá D. C Colombia; 2 Escuela de Salud Pública, Universidad del Valle, Santiago de Cali, Colombia Universidad del Valle Universidad del Valle Santiago de Cali Colombia; 3 Secretaría Distrital de Salud Pública, Santiago de Cali, Colombia Secretaría Distrital de Salud Pública Santiago de Cali Colombia; 4 Red Nacional de Investigación Innovación y Gestión del Conocimiento en Tuberculosis, Bogotá, D. C., Colombia Red Nacional de Investigación Innovación y Gestión del Conocimiento en Tuberculosis Bogotá D. C Colombia; 5 Grupo de Investigación Esculapio, Universidad Libre Seccional Cali, Santiago de Cali, Colombia Universidad Libre Universidad Libre Cali Colombia

**Keywords:** tuberculosis, control de infecciones, indicadores de desarrollo sostenible, administración de los servicios de salud, Colombia, Tuberculosis, infection control, sustainable development indicators, health services administration, Colombia

## Abstract

**Introducción.:**

El distrito de Santiago de Cali es la segunda entidad territorial con mayor número de casos de tuberculosis en Colombia. Desde el 2016, la Secretaría de Salud Pública implementó el plan de control de infecciones en las instituciones prestadoras de servicios de salud con programas activos contra la tuberculosis, lo que hizo necesaria su evaluación.

**Objetivo.:**

Contrastar los aspectos normativos y los elementos contextuales que influyen en la operación del plan de control de infecciones de tuberculosis, para documentar buenas prácticas y áreas de oportunidad en las instituciones prestadoras de servicios de salud.

**Materiales y métodos.:**

Se llevó a cabo un estudio evaluativo con enfoque mixto para analizar las capacidades institucionales del plan de control de infecciones. El componente cuantitativo incluyó la revisión documental y la evaluación de las medidas administrativas, ambientales y de protección respiratoria. Con el componente cualitativo, mediante el estudio de caso, se analizaron la normatividad, la gobernanza, el financiamiento, los sistemas de información, la infraestructura y la formación del personal. La información se integró mediante triangulación.

**Resultados.:**

El cumplimiento promedio del plan de control de infecciones fue del 70,8 % en las medidas administrativas, del 62,1 % en el control ambiental y del 90,5 % en de protección respiratoria. Se identificó la ausencia de normatividad nacional específica, frecuente rotación del personal, falta de capacitación, brechas en inversión y mantenimiento ambiental, e insuficiente monitoreo de la tuberculosis ocupacional. Entre las fortalezas, se destacaron la captación de pacientes sintomáticos respiratorios y la disponibilidad de elementos de protección respiratoria.

**Conclusiones.:**

Se evidenciaron brechas estructurales asociadas con la falta de un marco normativo nacional, debilidades de gobernanza, limitaciones financieras y deficiencias en el seguimiento y en la formación. Se recomienda determinar responsabilidades, asignar recursos y establecer protocolos operativos en las instituciones prestadoras de servicios de salud. La sostenibilidad del plan de control de infecciones requiere indicadores de seguimiento, auditorías periódicas y capacitación continua.

## Abstract (en)

**Introduction.:**

The district of Santiago de Cali is the second territorial entity with the highest number of tuberculosis cases in Colombia. Since 2016, the *Secretaría de Salud Pública* has implemented the infection control plan in health service provider institutions with active tuberculosis programs, making its evaluation necessary.

**Objective.:**

To contrast the regulatory aspects and contextual elements that influence the operation of the infection control plan for tuberculosis, in order to document good practices and areas for improvement in health service provider institutions.

**Materials and methods.:**

An evaluative study with a mixed-methods approach was conducted to analyze institutional capacities of the infection control plan. The quantitative component included document review and assessment of administrative, environmental, and respiratory control measures. The qualitative component -through a case study- analyzed regulations, governance, financing, information systems, infrastructure, and staff training. Data were integrated through triangulation.

**Results.:**

Average compliance of the infection control plan was 70.8% for administrative measures, 62.1% for environmental control, and 90.5% for respiratory health control. The study identified the absence of specific national regulations, high staff turnover, lack of training, gaps in investment and environmental system maintenance, and weak monitoring of occupational tuberculosis. Strengths included the identification of respiratory symptomatic individuals and the availability of respiratory protection equipment.

**Conclusions.:**

Structural gaps were identified, associated with the lack of a national regulatory framework, governance weaknesses, financial limitations, and deficiencies in monitoring and training. It is recommended to define responsibilities, allocate resources, and establish operational protocols in health service provider institutions. Ensuring the sustainability of the infection control plan requires monitoring indicators, periodic audits, and continuous training.

En Colombia, se notificaron 21 217 casos de tuberculosis (40,1 casos por cada 100 000 habitantes) en el 2024, de los cuales 306 (1,4 %) correspondieron a personal de salud ^1^. Desde el 2012, se ha observado un aumento progresivo de los casos de tuberculosis en el personal sanitario, con un crecimiento sostenido de 9 a 42 casos anuales ^2^.

Las personas que trabajan o reciben atención en los servicios de salud presentan mayores riesgos de infección por tuberculosis. Se ha indicado que el riesgo de presentar tuberculosis latente o de enfermar de tuberculosis es, aproximadamente, tres veces mayor en el personal sanitario comparado con la población general, lo que hace indispensable implementar un plan de control de infecciones como parte de un programa institucional ^3^^,^^4^. Este plan debe enfocarse en la detección temprana de casos, la aplicación de precauciones para la transmisión aérea y el tratamiento oportuno de las personas con diagnóstico presuntivo o confirmado ^5^.

De acuerdo con las recomendaciones de la Organización Mundial de la Salud (OMS), la Organización Panamericana de la Salud (OPS) y los *Centers for Disease Control and Prevention* (CDC), los programas de control de infecciones de la tuberculosis deben estructurarse en tres niveles complementarios:

1. medidas administrativas (triaje respiratorio, diagnóstico oportuno, aislamiento y seguimiento de personas con diagnóstico presuntivo o confirmado);
2. medidas de control ambiental (ventilación natural o mecánica, presión negativa, control de flujos de aire), y
3. protección respiratoria del personal (uso adecuado de respiradores y programas de ajuste y capacitación) ^6^^-^^8^.

El distrito de Santiago de Cali es la segunda entidad territorial del país que aporta el mayor número de casos de tuberculosis en Colombia. Para el 2016, la Secretaría de Salud Pública Municipal de Cali inició la implementación del plan de control de infecciones para las enfermedades transmitidas por vía aérea, con énfasis en la tuberculosis en las instituciones prestadoras de servicios de salud (IPS) como una estrategia prioritaria para detener la transmisión de la enfermedad. No obstante, su implementación ha presentado limitaciones estructurales que comprometen su sostenibilidad y efectividad. Además, la emergencia por SARS-CoV-2 evidenció con mayor claridad las debilidades del sistema.

En este sentido, el fortalecimiento del plan de control de infecciones exige una visión sistémica que permita consolidar la institucionalidad y una política pública sólida y sostenible, capaz de perdurar más allá de los ciclos administrativos o los cambios de personal.

Este estudio tuvo como propósito contrastar los aspectos normativos y los elementos contextuales de la operación del plan de control de infecciones con el fin de documentar las buenas prácticas y las áreas de oportunidad de la operación. La evaluación permitió evidenciar el cumplimiento de la implementación de las medidas de control, determinando si el diseño normativo facilita o dificulta el control de la tuberculosis en las IPS.

## Materiales y métodos

Entre julio del 2020 y agosto del 2022, se llevó a cabo un estudio evaluativo con enfoque mixto en 37 IPS de Cali, recolectando datos cuantitativos y cualitativos sobre las capacidades institucionales para implementar el plan de control de infecciones en tuberculosis.

Para el componente cuantitativo, se utilizó el marco lógico para evaluar el diseño del plan de control de infecciones de la Secretaría de Salud de Cali (2016-2019), realizando una revisión documental y analizando objetivos, problemas y aspectos técnicos, normativos y contextuales. La gestión operativa se examinó mediante la aplicación de una encuesta que permitió valorar los procesos administrativos, ambientales y de prevención en protección respiratoria; esta encuesta fue elaborada según las guías internacionales y locales, revisada por especialistas y validada en una IPS externa ^6^^-^^8^.

En el estudio cualitativo, se adoptó un diseño de tipo estudio de caso, de carácter exploratorio e interpretativo. En la unidad de análisis, se incluyeron las capacidades en salud pública dentro del plan de control de infecciones, con énfasis en tuberculosis, tomando como casos plan de control de infecciones de Cali. Se valoró el cumplimiento de los tres componentes operativos del plan de control de infecciones (administrativo o gerencial, ambiental y protección respiratoria), así como de factores estructurales -normativos, institucionales, financieros, técnicos, de evaluación y de formación- que influyeron positiva o negativamente en su sostenibilidad ^9^^-^^10^.

### Estrategia metodológica

#### 1. Fase cuantitativa

Se invitaron a participar a las 55 IPS de Cali que brindaban servicios de diagnóstico, tratamiento y seguimiento a pacientes con tuberculosis y que habían implementado el plan de control de infecciones entre el 2016 y el 2020. Todas las IPS se encontraban habilitadas y en funcionamiento durante el 2021.

Treinta y siete instituciones aceptaron participar en el estudio y permitieron la visita y la aplicación de la encuesta. La recolección de la información se llevó a cabo mediante visitas presenciales entre octubre del 2021 y enero del 2022, periodo en el cual persistían restricciones debidas a la pandemia por SARS-CoV-2.

La gerencia de cada IPS asignó a un responsable de responder la encuesta: en el 41 % (15/37) de los casos respondió el encargado del comité de infecciones o vigilancia epidemiológica; en el 35 % (13/37), el jefe de infectología; en el 14 % (5/37), el encargado del programa de tuberculosis; y, en el 11 % (4/37), el enfermero jefe junto al equipo de ingeniería.

Según el nivel de complejidad y la naturaleza jurídica, las IPS se clasificaron en: 17 primarias públicas, 3 primarias privadas, 14 complementarias privadas y 3 complementarias públicas.

#### 2. Fase cualitativa

Se seleccionaron 5 IPS por conveniencia y se evaluaron seis dimensiones de las capacidades en salud pública:

1. normativo-institucional: existencia de directrices internas, funciones delegadas, comités activos;
2. gobernanza y fuerza laboral: liderazgo, compromiso gerencial, estabilidad del talento humano;
3. sistemas de información: uso, acceso y calidad de los datos relacionados con el plan de control de infecciones;
4. financiamiento: fuentes y uso de recursos específicos para el plan de control de infecciones;
5. infraestructura y tecnología: condiciones físicas, ventilación, señalización, flujos de aire para la prevención de la transmisión, y
6. formación y cultura organizacional: frecuencia y énfasis de los procesos de capacitación.

Además, se adelantó la revisión de la normativa nacional y de las asistencias técnicas realizadas por la autoridad sanitaria a los planes institucionales de control de infecciones. Se conformaron cinco grupos focales con informantes clave, con la asistencia de seis personas como máximo, incluyendo personal asistencial, de laboratorio clínico, coordinadores de servicios, personal de seguridad y servicios generales. La información fue transcrita, categorizada y analizada.

### Análisis de datos

*Cuantitativo:* los datos cuantitativos de las 37 instituciones participantes fueron analizados mediante estadística descriptiva utilizando Stata 16™ (Stata Corp, Collage Station, TX, USA). Las variables se organizaron por distribución de frecuencias absolutas y relativas. Para la interpretación de los niveles de cumplimiento del plan de control de infecciones, se aplicó el instrumento de evaluación de la Secretaría de Salud Pública Municipal del 2016, a saber, una escala de tipo semáforo establecida institucionalmente (verde ≥ 80 %, amarillo 60 a 79 %, rojo ≤ 59 %).

*Cualitativo:* la información obtenida se procesó mediante un análisis de contenido. En primer lugar, se realizó una codificación abierta para desglosar los textos y generar los conceptos iniciales. Estos conceptos se organizaron posteriormente mediante una codificación axial, agrupándolos en categorías y subcategorías con las dimensiones predefinidas, lo que permitió explorar sistemáticamente las relaciones (contexto, condiciones, interacciones) dentro de las categorías y entre ellas, con el objetivo de identificar patrones narrativos dominantes. El análisis se orientó a comprender los factores contextuales, determinantes y condicionantes de dichas relaciones, así como sus efectos y consecuencias en cada institución (caso de estudio). Este abordaje facilitó una explicación de los logros y de las limitaciones en la implementación del plan de control de infecciones en las IPS.

*Triangulación:* para fortalecer los hallazgos y garantizar su validez, se integraron los resultados de ambas fases del estudio (cuantitativa y cualitativa), lo que permitió contrastar y comparar los niveles de cumplimiento cuantificados con las interpretaciones cualitativas sobre los factores operativos y estructurales subyacentes.

La síntesis se materializó en la construcción de una matriz DOFA (debilidades, oportunidades, fortalezas, amenazas), la cual sirvió como herramienta analítica para cruzar de manera estructurada las categorías y subcategorías identificadas con el grado de alcance de los indicadores del plan de control de infecciones. Esta matriz fue validada en una sesión de trabajo con actores institucionales, asegurándose la pertinencia y credibilidad del análisis.

El proceso de triangulación permitió identificar convergencias y divergencias de los datos, revelando patrones integrales y relaciones contextuales entre las dimensiones internas (fortalezas o debilidades) y externas (oportunidades o amenazas) que influyen en la implementación del plan de control de infecciones.

### Aspectos éticos

El estudio fue aprobado por el Comité de Ética y Metodologías en Investigación del Instituto Nacional de Salud (CEMIN-10-2019). Se garantizó la confidencialidad de la información y se obtuvo el consentimiento informado de las instituciones y de los profesionales participantes.

## Resultados

Las 37 instituciones analizadas presentaron un promedio del 70,8 % en el cumplimiento de la implementación de las medidas administrativo-gerenciales, el 62,1 % para las medidas de control ambiental y el 90,5 % para las medidas de protección respiratoria, según las actividades evaluadas del plan de control de infecciones de tuberculosis (cuadro 1).

**Cuadro 1:** Triangulación de la información de la evaluación de la implementación de las medidas del plan de control de infecciones para la tuberculosis, Cali, 2019-2021

Componentes	Variables	Semaforización (%)
Medidas de control administrativo	1. Organización, elaboración del plan de control de tuberculosis, evaluación del riesgo y entrenamiento	76,7
	2. Triaje, separación y tratamiento oportuno	83,2
	3. Vigilancia de la enfermedad tuberculosa en trabajadores de salud	52,6
	Calificación global	70,8
Medidas de control ambiental	1. Uso de elementos para control ventilatorio	27,3
	2. Mantenimiento de equipos e infraestructura para la protección respiratoria	75,2
	3. Mejora del sistema ventilatorio	83,7
	Calificación global	62,1
Medidas de protección respiratoria	1. Contar con un programa escrito de protección respiratoria	91,8
	2. Entrenamiento a los trabajadores en protección respiratoria	89,1
	3. Poseer una guía o protocolo para el uso de respiradores N95 y mascarillas quirúrgicas	91,8
	4. Hacer seguimiento al uso de los equipos de protección respiratoria	97,2
	5. Realizar prueba de ajuste de los respiradores N-95 en los trabajadores	75,6
	6. Los respiradores N-95 estaban certificados por NIOSH u OSHAS.	97,2
	Calificación global	90,5

Verde: cumplimiento del 80 al 100 %; amarillo: cumplimiento del 60 al 79 %; rojo: menor del 59 %

Los resultados detallados de las encuestas han sido publicados y se encuentran disponibles para su consulta ^11^.

### Componente normativo-institucional

Se identificaron tres categorías: políticas sanitarias, control de infecciones de tuberculosis y políticas relacionadas con la seguridad y la salud en el trabajo.

En el ámbito de las políticas sanitarias no se encontró normatividad específica para el plan de control de infecciones de tuberculosis. Se revisaron los ocho documentos nacionales que han sustentado la organización del sistema de salud y los requisitos de calidad para las IPS, incluidas las normas que establecen las medidas sanitarias generales, ambientales, ocupacionales y de protección personal (Ley 9 de 1979); las que establecen las condiciones sanitarias para las edificaciones hospitalarias -incluyendo aislamiento (Resolución 4445 de 1996)-; las referidas a las auditorías y estándares de calidad -incluyendo las infecciones intrahospitalarias (Decreto 1 011 de 2006 y Resolución 1043 de 2006)- y las que refuerzan la habilitación en la presentación de los servicios con énfasis en bioseguridad y educación del personal (Resolución 2003 de 2014).

Para el marco normativo específico en control de infecciones y tuberculosis, el Ministerio de Salud y Protección Social en Colombia cuenta con el *Plan estratégico hacia el fin de la tuberculosis 2016-2025,* en el que se propone asegurar el plan de control de infecciones en el 100 % de las entidades territoriales, promover comités de control y adoptar protocolos de bioseguridad.

El *Manual de medidas básicas para el control de infecciones en IPS* incluye una adaptación de los estándares internacionales del control de infecciones y de las guías para el aislamiento y el uso de elementos de protección personal.

Finalmente, el marco relacionado con la salud ocupacional reconoce la tuberculosis como riesgo laboral y dicta la responsabilidad del empleador en la prevención de la tuberculosis como enfermedad laboral. No obstante, la articulación con salud pública es débil, pues ninguno de los dos documentos decretados para el manejo de estos riesgos (Decreto 1477 de 2014 y Decreto 1072 de 2015) presenta una articulación operativa o financiera con el *Programa de prevención y control de tuberculosis.*

Con relación al cumplimiento en la implementación del plan de control de infecciones desde el punto de vista normativo e institucional, la mayoría de las IPS analizadas (86,4 %) (32/37) no contaban con una evaluación del riesgo relacionada con la transmisión de enfermedades por vía aérea, como la tuberculosis. La evaluación del funcionamiento de los comités de control de infecciones reveló que, en general, las acciones específicas para el control de la tuberculosis no son asumidas por estos comités, sino por los comités de vigilancia epidemiológica, lo que dificulta la articulación con otros departamentos clave, como mantenimiento e ingeniería mecánica (para el control ambiental), salud ocupacional (gestión del riesgo laboral) y compras (garantía de elementos de protección personal). Se evidenció que los comités carecían de indicadores de monitoreo y evaluación, así como de mecanismos efectivos de retroalimentación y de comunicación gerencial.

Frente a las acciones del plan de control de infecciones en los laboratorios clínicos, las instituciones reportaron que la adopción de políticas públicas desde el nivel gerencial facilitó la implementación de protocolos de bioseguridad y transporte de muestras. Aunque la mayoría de las instituciones terceriza el servicio de laboratorio para el diagnóstico de la tuberculosis, se han adquirido tecnologías que reducen la exposición del personal a la tuberculosis durante la recepción, el almacenamiento y la remisión de muestras.

Todas las instituciones cumplían con los procesos de desinfección de espacios y equipos requeridos por las normas para la habilitación de servicios, incluyendo el suministro de insumos como jabón, soluciones antibacterianas, estructuras para el lavado de manos y toallas de papel, así como lo indica un profesional en el grupo focal:

«[...] A mí me parece que el comité de infecciones ha sido muy completo en el tema desde gestión ambiental, el tema de limpieza y desinfección para nosotros es supremamente importante (...). Nos encargamos de las superficies hospitalarias que son las que más interfieren en el tema de infecciones. Trabajamos con luminometrías, tenemos una empresa tercerizada que se hace cargo de la limpieza y desinfección; aparte de eso, tenemos las supervisoras y todo el tiempo se está retroalimentando, se está capacitando y se está evaluando el contrato con ellos y la labor que hacen aquí en la institución [...]» (profesional en salud de la institución 4, 2021).

En cuanto a las políticas y normas institucionales, el 91,8 % (34/37) de las instituciones contaba con programas escritos de protección respiratoria y con protocolos de bioseguridad reportados en sus procesos de habilitación. La protección respiratoria, en particular, se generalizó al uso de mascarillas quirúrgicas y respiradores N95 para el personal sanitario en las áreas de mayor riesgo, como las unidades de cuidados intensivos y los programas de atención de pacientes con tuberculosis.

### Componente de gobernanza y fuerza laboral

Aunque fue priorizada la atención de pacientes sintomáticos respiratorios en las IPS, solo el 56,7 % (21/37) contó con seguimiento, principalmente por la falta de personal exclusivo y su frecuente rotación. De igual manera, entre la fuerza de trabajo se pudo evidenciar -por parte de algunos profesionales-desconocimiento de las directrices del plan de control de infecciones en sus tres componentes. Esto se relacionó con la frecuente rotación de personal y la ausencia de procesos de formación continua, lo cual fue referido por un profesional en el grupo focal:

«[...] Las capacitaciones no eran suficientes en ese momento, en esa medida las personas no tenían conciencia suficiente de que deben usar tapabocas, ni siquiera en el laboratorio usaban tapabocas; uno usaba tapabocas en el laboratorio cuando ya iba a montar las muestras [...]» (profesional en salud de la institución 2, 2021).

### Componente financiero

Las IPS han realizado esfuerzos y han invertido recursos para mejorar las condiciones arquitectónicas institucionales. El 81 % (30/37) de las instituciones mostró voluntad de la gerencia para invertir en rediseños arquitectónicos que permitieran contar con cuartos de aislamiento para los pacientes sintomáticos respiratorios en urgencias y en hospitalización; sin embargo, se observaron deficiencias en los estudios técnicos relacionados con el cumplimiento de los flujos de aire y de presión negativa. En este sentido, a pesar de que el 67,5 % (24/37) de las instituciones refirió procesos de conocimiento de las técnicas y seguimiento a los flujos de aire, no se evidenciaron procesos de monitoreo o evaluación o en la toma de decisiones desde los planes de mejora.

Por otro lado, se observó un mayor cumplimiento en la disposición de consultorios exclusivos para los pacientes con tuberculosis y en el distanciamiento de las camas en hospitalización. A pesar de estos esfuerzos, persisten limitaciones en infraestructura, como la falta de espacios para la recolección de las muestras de esputo.

La alta calificación en el cumplimiento de las acciones del componente ambiental frente a la tenencia de sistemas ventilatorios institucionales, no estuvo relacionada con los estudios de riesgos de exposición ocupacional o del monitoreo de los flujos de aire que permitieran la implementación de estas estrategias en aquellas áreas con mayor riesgo de transmisión.

Los controles implementados en los laboratorios clínicos que mostraron un proceso de transición hacia nuevas tecnologías para disminuir la exposición a agentes patógenos de transmisión por vía aérea que es uno de los avances destacados en este componente, junto con los rediseños arquitectónicos a cargo de expertos vinculados a los proveedores de las tecnologías.

Respecto a la inversión en protección respiratoria, todas (37) las instituciones contaban con stock de respiradores N95, aunque para algunas instituciones el uso de estos estaba restringido a que el respirador cumpliera con la reglamentación requerida de NIOSH u OSHAS. Los departamentos de compras eran los únicos encargados de la verificación del cumplimiento de estos requisitos, por lo que esta información era desconocida por el personal asistencial que usa los respiradores, quienes solo sabían que había una disponibilidad permanente de los productos:

«[...] Por lo general se maneja la matriz de elementos de protección personal, donde se identifica la cantidad que les corresponden dependiendo de la exposición, eso es lo primero. Ya posterior se definen los elementos de protección personal con el proveedor, se solicitan fichas técnicas y se realiza la entrega de los elementos de protección personal dependiendo de la frecuencia, pues que se haya definido. Según la matriz se deja registro de la entrega; ese lo consolidamos de manera semanal para tener orden y la evidencia [...]» (profesional en salud de la institución 4, 2021).

### Componente técnico-operativo

El 83,2 % de las instituciones mostró un buen desempeño en la implementación de acciones para frenar la cadena de transmisión de la tuberculosis. Una de las estrategias más relevantes fue la implementación de rutas para el manejo de los pacientes sintomáticos respiratorios en el 91,8 % de las IPS. En el 89,1 % se desarrollaron estrategias de captación mediante piezas de información, educación y comunicación dirigidas a profesionales y visitantes en las salas de espera y los consultorios. Estas acciones permitieron priorizar a los usuarios en el triaje, aunque se identificaron vacíos en servicios de gran afluencia, como pediatría, ginecobstetricia y odontología.

El 86,4 % (32/37) de las instituciones hizo seguimiento al cumplimiento de las condiciones ambientales, principalmente en lo que respecta a los requisitos para habilitar el funcionamiento de los servicios. Las tecnologías para la limpieza del aire se ubicaron principalmente en las unidades de cuidados intensivos, cuartos de hospitalización y consultorios de aislamiento. No obstante, 21 de las 37 instituciones reportaron desconocimiento por parte del personal de la existencia y utilidad de estos sistemas. No se identificaron procesos de mantenimiento rutinarios. Así lo indicaron en un grupo focal:

«[...] Sí tenemos la infraestructura, pero, que haya funcionado como tal, no. Tenemos los cuartos de aislamiento en hospitalización, no digamos un cuarto como para la utilización para la terapia respiratoria, pero, presencia de filtro HEPA como tal en la institución, no […]» (profesional en salud de la institución 3, 2021).

### Componente de evaluación y monitoreo

No se encontraron indicadores de monitoreo y evaluación en los tres componentes del plan de control de infecciones (administrativo o gerencial, ambiental y de protección respiratoria), como tampoco, en la retroalimentación al personal. La evaluación y el seguimiento de los casos de tuberculosis entre los trabajadores institucionales presentaron un desempeño deficiente; solo el 52,6 % de las instituciones implementó acciones de vigilancia en tuberculosis entre los profesionales de salud.

Para el 2019, el 62,1 % (23/37) de las organizaciones registró casos de tuberculosis en su personal sanitario; el resto de las IPS delegaba esta responsabilidad a la institución notificadora o a la institución prestadora de servicios de salud en la cual se encontraba asegurado el trabajador, así como se evidencia en un grupo focal:

«[...] Actualmente, cuando identifico algún colaborador que había estado en riesgo con algún caso positivo para tuberculosis, inmediatamente se hace la comunicación con vigilancia epidemiológica y comité de infecciones, y entre los dos inician un seguimiento de estos síntomas y de contactos estrechos; en ese momento se direcciona a su IPS o aquí a la institución en caso de que corresponda para hacer los respectivos exámenes, entonces pienso que, de pronto, eso no se hacía en 2019 y ahora pues ha dado buenos resultados […]» (profesional en salud de la institución 4, 2021).

Solo el 43,2 % (16/37) de las instituciones realizó seguimiento del estado de salud del personal mediante la prueba de tuberculina, herramienta clave para evaluar el riesgo de infección. La aplicación de esta prueba se limitó a los profesionales considerados con mayor riesgo de exposición, debido a una insuficiente articulación entre el área de seguridad y salud en el trabajo y los programas de vigilancia de tuberculosis. A esto se suma la falta de acceso rutinario a la prueba para los trabajadores asistenciales, administrativos, de servicios generales y de vigilancia; el desabastecimiento nacional de la PPD durante el periodo de observación, y la ausencia de reportes ocupacionales de accidentes o enfermedades que activen los procesos de investigación de eventos con posible origen laboral.

A pesar de lo anterior, una institución refiere practicar la prueba de tuberculina:

«[...] A todas las personas de la institución se les pide una tuberculina; en caso de ser negativa, se sigue solicitando todos los años; y, en el caso de ser positiva, se pasa con el área de salud ocupacional […]» (profesional en salud de la institución 5, 2021).

Para el componente de control de la protección respiratoria se presentó controversia entre los resultados cuantitativos y los cualitativos, en los que, a pesar de que se reporta un cumplimiento del 97,2 % (36/37), el personal refirió fallas en la adopción de las medidas de protección, incluidas la formación del personal y la falta de procesos de monitoreo en el uso adecuado de los respiradores N95.

### Componente de formación y cultura organizacional

Respecto a la formación del personal, el 97,2 % (36/37) de las instituciones ofreció capacitación continua anual, centrada exclusivamente en el cuidado respiratorio, sin incluir los temas de control administrativo y ambiental que influencian la transmisión de la tuberculosis. En el 62 % (23/37) se excluyó al personal administrativo, de servicios generales y de seguridad, a pesar de su exposición. Estas capacitaciones buscaban fortalecer el cuidado respiratorio e incorporarlo en las rutinas laborales diarias para la disminución del riesgo de tuberculosis, enfocado en el uso adecuado de los elementos de protección personal, así como lo indica un profesional en el grupo focal:

«[…] nosotros en lo que más invertimos es en la norma de bioseguridad. todo lo relacionado a este tema lo hacemos de manera didáctica en los servicios para que sea como un tema más amigable […]» (profesional en salud de la institución 1, 2021).

No obstante, no se evidenciaron sistemas de información o evaluación que permitieran verificar el conocimiento adquirido ni la observancia de las recomendaciones impartidas.

A pesar de los avances en las mediciones reportadas de flujos de aire, en 21 de 37, los profesionales desconocen al departamento y al personal responsable de su implementación, seguimiento y monitoreo. Esto evidencia una desarticulación entre los procesos de evaluaciones técnicas del plan de control de infecciones y las acciones rutinarias del personal asistencial en las áreas de riesgo.

Una de las manifestaciones reiteradas entre los profesionales institucionales, es la falta de socialización frente a la necesidad de ubicación y acción de los flujos de aire y de los seguimientos de las mediciones que permita la adopción de acciones individuales frente en los espacios de trabajo, así como lo expresa un profesional en el grupo focal:

«[...] Yo estuve en el laboratorio clínico realizando baciloscopias durante varios años y no se maneja un seguimiento. A mí y a mis compañeras jamás nos han hecho un seguimiento [...]» (auxiliar asistencial de la institución 2, 2021).

Como parte de la organización anual de los programas de mantenimiento, se observó la existencia de un plan de mantenimiento preventivo en el 91,8 % (34/37) de las instituciones con ventilación mecánica, condición que se refleja en menor medida en las instituciones con ventilación natural [89,1 %, (33/37)].

De manera generalizada, el personal asistencial desconoce los procesos de mantenimiento de la ventilación, refiriendo que dichos mantenimientos son realizados en horario diferente al de la prestación de sus servicios, sumado a la baja percepción del riesgo que puede afectar el estado actual o futuro de su salud.

El 81,9 % de las instituciones cuenta con un entrenamiento anual sobre protección respiratoria que incluye la forma y el tiempo de uso de los implementos de protección, así como su almacenamiento adecuado; algunas instituciones priorizaron el uso de mascarillas quirúrgicas para profesionales asistenciales.

## Discusión

Los hallazgos del presente estudio confirman que la implementación del plan de control de infecciones con énfasis en la tuberculosis de las IPS de Cali enfrenta obstáculos significativos, particularmente respecto a la consolidación de los componentes estructurales que garanticen su sostenibilidad y adaptación institucional. Si bien los niveles de cumplimiento para las medidas de protección respiratoria y las acciones de mejora del sistema de ventilación fueron altos, los déficits en planificación, articulación interdepartamental, monitoreo y formación revelan una débil institucionalización del plan de control de infecciones como política pública efectiva.

Houghton y colaboradores evidenciaron la relevancia de incorporar las capacidades en salud pública como factores clave para la observación durante la implementación del plan de control de infecciones, considerando sus tres componentes principales ^12^. En su estudio, identificaron ocho subdominios destinados a analizar los factores que inciden en la observancia del plan de control de infecciones.

En el ámbito del control administrativo, se reconocieron como factores organizativos la seguridad, la existencia de programas específicos de seguridad y salud ocupacional, así como la oferta de programas de formación. Respecto al control ambiental, se subrayó la importancia del entorno físico y la disponibilidad de tecnologías de protección.

Para el componente de protección respiratoria, se identificaron factores individuales tales como el conocimiento, la actitud y las creencias. Con base en estos hallazgos, las evaluaciones del plan de control de infecciones no deben limitarse solo al cumplimiento en la implementación, sino que también, deberían abordarse los conocimientos, actitudes y prácticas del personal ^13^^,^^14^. Por lo tanto, restringirse únicamente a las prácticas individuales recomendadas puede resultar insuficiente para evaluar su efecto ^15^. En consecuencia, el abordar el seguimiento bajo la perspectiva de mejoramiento continuo, conforme al modelo de entorno laboral saludable, facilitaría considerar el plan de control de infecciones tanto en su desarrollo como en su impacto dentro del contexto de la IPS.

Desde el componente normativo e institucional, la ausencia de una evaluación del riesgo en la mayoría (32/37) de las IPS limita el enfoque preventivo y la priorización de intervenciones hacia áreas de mayor riesgo, lo que puede llevar a la inversión inadecuada de recursos. Esta falencia evidencia que el plan de control de infecciones sigue siendo percibido como un requisito para la habilitación de los servicios de salud que acarrea sanciones administrativas en caso de no cumplirse, en vez de percibirse como una estrategia integral institucional. Esto impide relacionar adecuadamente el nivel de riesgo con factores clave como la carga de la enfermedad según la ubicación geográfica, el tipo de tuberculosis y las características de vulnerabilidad de los usuarios. En consecuencia, no se implementan acciones adaptadas al contexto.

El riesgo anual de infección tuberculosa ocupacional en los hospitales ubicados en las zonas de gran incidencia oscila entre el 1,0 y el 5,0 %, siendo mayor que el riesgo en la comunidad ^4^^,^^16^. Este riesgo está influenciado por factores como la implementación de las medidas adecuadas de control de las infecciones en las instituciones de salud, la evaluación del riesgo por áreas y por procedimientos realizados (siendo mayores en aquellos que generan aerosoles), además de la variación inherente a la composición étnica y demográfica del personal ^17^^,^^18^.

Los resultados del presente estudio demostraron debilidades institucionales de fuerza laboral con frecuentes rotaciones del personal de salud que influyen en el desconocimiento de las directrices y políticas institucionales de bioseguridad y control de infecciones.

Las medidas del plan de control de infecciones son muy efectivas para reducir la transmisión de tuberculosis y pueden adoptarse fácilmente en los centros de salud con recursos limitados; sin embargo, es fundamental que se implementen junto con otras estrategias como la educación del paciente, la cultura laboral que genere compromiso del trabajador, la disponibilidad de elementos de protección personal, el suministro adecuado de respiradores y de instalaciones de aislamiento adecuadas ^18^^-^^20^.

La integración de las acciones del plan de control de infecciones dentro del comité de vigilancia epidemiológica en lugar del comité de control de infecciones, del cual debe hacer parte el director o su delegado, refleja una débil institucionalidad que impide la coordinación entre áreas técnicas clave (ingeniería, compras, seguridad y salud en el trabajo). Esta falta de estructura formal limita la planeación, el seguimiento y la mejora continua, especialmente en el componente ambiental, el más rezagado en su implementación.

Medidas sencillas como identificar la tos, aislar pacientes, ventilar espacios y usar mascarillas, son efectivas para reducir la transmisión de la tuberculosis y pueden aplicarse fácilmente, incluso con recursos limitados, si se acompañan de educación, cultura de apoyo y uso adecuado de los elementos de protección personal ^21^.

Parte de la cultura laboral implica la asignación de tareas, responsabilidades y cargas laborales frente a la ejecución del plan de control de infecciones; en ellas deben tenerse en cuenta las funciones de los comités institucionales que parten de la planeación, la ejecución y el monitoreo.

Desde el componente técnico y operativo, las instituciones contaban con prácticas de captación y aislamiento de los pacientes sintomáticos respiratorios y de educación del paciente. Estas prácticas hacen parte de las medidas del control administrativo que son la primera línea de acción y deben priorizarse, ya que el objetivo es prevenir la exposición del personal y de los pacientes a la tuberculosis mediante la captación, el diagnóstico y el tratamiento oportuno ^22^. Sin embargo, debe garantizarse la implementación y el funcionamiento correcto de todos los componentes del sistema, incluida la gobernanza y la administración, la financiación, la infraestructura, las adquisiciones y los suministros, los recursos humanos, los sistemas de información y de vigilancia sanitaria, la prestación de servicios, la supervisión y la toma de decisiones basadas en la evidencia.

Respecto a los sistemas de información, los estudios destacan la importancia de contar con informes de implementación de medidas como, por ejemplo, el triaje para los pacientes con tuberculosis pulmonar presuntiva, los registros de ventanas abiertas, las actas de las reuniones del comité de control de infecciones, los registros de la participación en capacitaciones sobre el plan de control de infecciones y las pruebas de ajuste de los elementos de protección respiratoria ^23^. En las IPS incluidas en el estudio, no se contaba con un sistema de información; por tanto, no fue posible realizar un seguimiento y una evaluación del plan de control de infecciones. Esto probablemente estaba relacionado con el hecho de que no se contaba con indicadores definidos.

Muchas falencias se evidenciaron en el monitoreo y en la supervisión, en parte debidas a la falta de vigilancia epidemiológica de los casos de tuberculosis entre los trabajadores de salud, y al conocimiento y mantenimiento de los equipos de ventilación. Para el 62 % (23/37) de las instituciones, dentro de los procesos de formación no se vinculaba al personal administrativo, de servicios generales, mantenimiento y vigilancia, lo cual imposibilita el desarrollo de la capacidad de autocuidado del personal expuesto a la micobacteria, a lo que se suma el desconocimiento sobre las condiciones personales que aumentan el riesgo de exposición en el ambiente de trabajo a la tuberculosis. Al abordar el comportamiento del personal frente a las medidas del plan de control de infecciones, se hace necesario considerar las creencias y percepciones de riesgo de tuberculosis, el cual está influido por el contexto social, político y normativo en el que se implementa el plan de control de infecciones ^24^. Aunado a esto, se ha evidenciado mayor observancia cuando las capacitaciones se han orientado al desarrollo de habilitades en el personal ^20^.

En conclusión, la implementación del plan de control de infecciones en tuberculosis en las IPS de Cali presenta importantes brechas estructurales que limitan su sostenibilidad y eficacia. Aunque existen buenas prácticas en algunas instituciones prestadoras de servicios de salud, estas dependen de esfuerzos individuales más que de estructuras sólidas o decisiones políticas.

Los componentes financieros, normativo-institucional y de monitoreo y evaluación, tuvieron los menores cumplimientos, lo que refleja un bajo nivel de institucionalización del plan de control de infecciones como política pública local. La normatividad existente no garantiza la implementación real del plan, lo que plantea el reto de fortalecer el marco político-administrativo para lograr la implementación, la sostenibilidad y el monitoreo.

Se recomienda fortalecer el plan de control de infecciones mediante su incorporación como línea estratégica en el plan distrital de la Secretaría de Salud y en la normatividad interna de las IPS, garantizando su integración con otros planes institucionales. Se deben designar responsables formales del plan de control de infecciones, reactivar los comités de control de infecciones y consolidar la gobernanza distrital en la que se mantenga el monitoreo y control constantes.

En el ámbito financiero, se sugiere asignar presupuestos específicos y gestionar recursos para las adecuaciones y la capacitación basada en la evidencia de desconocimiento por parte del personal de salud. Operativamente, se deben establecer protocolos técnicos mínimos y fortalecer la coordinación interdepartamental en las IPS.

En evaluación y seguimiento, es importante incluir indicadores del plan de control de infecciones en el sistema de monitoreo y llevar a cabo auditorías periódicas para orientar las decisiones. Finalmente, se recomienda implementar un plan distrital de formación continua y promover una cultura organizacional centrada en la prevención, con incentivos y reconocimiento a las buenas prácticas, garantizando la sostenibilidad y efectividad del plan de control de infecciones en las IPS.

Este estudio presentó algunas limitaciones que deben considerarse en la interpretación de los resultados. En primer lugar, la participación voluntaria de 37 de las 55 IPS elegibles puede introducir sesgo de selección y limitar la representatividad de los hallazgos. La recolección de información durante la pandemia por SARS-CoV-2 pudo influir en la priorización institucional de acciones y en el cumplimiento de las medidas de protección respiratoria no debidas a la protección de tuberculosis.

La fase cualitativa se realizó en una muestra por conveniencia de cinco IPS, lo que limita la transferibilidad de los hallazgos. Finalmente, el desabastecimiento nacional de tuberculina y la deficiente articulación entre los programas de tuberculosis y de seguridad y salud en el trabajo, pudieron afectar la valoración del componente de vigilancia ocupacional y el posible subregistro de casos en personal sanitario.
